# Fractional Time Fluctuations in Viscoelasticity: A Comparative Study of Correlations and Elastic Moduli

**DOI:** 10.3390/e20010028

**Published:** 2018-01-11

**Authors:** Rosalío F. Rodríguez, Elizabeth Salinas-Rodríguez, Jorge Fujioka

**Affiliations:** 1 Instituto de Física, Universidad Nacional Autónoma de México, Apdo. Postal 20-364, Cd. de México 01000, Mexico; 2 Sistema Nacional de Investigadores, Mexico City 03940, Mexico; 3 Proyecto Universitario de Fenómenos Lineales y Mecánica (FENOMEC), Universidad Nacional Autónoma de México, Apdo. Postal 20-364, Cd. de México 01000, Mexico; 4 Departamento I. P. H., Universidad Autónoma Metropolitana, Iztapalapa, Apdo. Postal 55-534, Cd. de México 09340, Mexico

**Keywords:** fluctuations, elastic moduli, correlation functions

## Abstract

We calculate the transverse velocity fluctuations correlation function of a linear and homogeneous viscoelastic liquid by using a generalized Langevin equation (*GLE*) approach. We consider a long-ranged (power-law) viscoelastic memory and a noise with a long-range (power-law) auto-correlation. We first evaluate the transverse velocity fluctuations correlation function for conventional time derivatives ${\hat{C}}_{NF}\left(\vec{k},t\right)$ and then introduce time fractional derivatives in their equations of motion and calculate the corresponding fractional correlation function. We find that the magnitude of the fractional correlation ${\hat{C}}_{F}\left(\vec{k},t\right)$ is always lower than the non-fractional one and decays more rapidly. The relationship between the fractional loss modulus $G_{F}^{\prime \prime }\left(\omega \right)$ and ${\hat{C}}_{F}\left(\vec{k},t\right)$ is also calculated analytically. The difference between the values of $G^{\prime \prime }\left(\omega \right)$ for two specific viscoelastic fluids is quantified. Our model calculation shows that the fractional effects on this measurable quantity may be three times as large as compared with its non-fractional value. The fact that the dynamic shear modulus is related to the light scattering spectrum suggests that the measurement of this property might be used as a suitable test to assess the effects of temporal fractional derivatives on a measurable property. Finally, we summarize the main results of our approach and emphasize that the eventual validity of our model calculations can only come from experimentation.

## 1. Introduction

The prominent role that time correlation functions have played in the description of non-equilibrium properties of fluids stems from their close connection with their transport coefficients, a relation that can be obtained from time-dependent correlation functions of suitable fluxes [1]. In a simple fluid in near-to-equilibrium states the central limit theorem (*CLT*) applies and a well-defined separation between the time scales associated with the macroscopic transport processes and the microscopic processes giving rise to them exists. As a consequence, the stochastic dynamics of the thermal fluctuations around equilibrium can be characterized as a Gaussian–Markovian process [2,3,4,5]. However, the presence of long noise-correlation or long time-memory in the time evolution equations for the fluctuations may destroy this separation, and the usual description of fluctuations in terms of the conventional Langevin equations may no longer be adequate [6,7,8,9,10,11]. This situation may occur in a large variety of relaxation processes in complex systems like viscoelastic fluids, glassy materials, synthetic polymers or biopolymers, all of which have in common that their relaxation functions are non-exponential, due to the large number of highly coupled elementary units responsible for the relaxation. As a consequence, the *CLT* is not applicable and the requirement of a high cooperation between these elements leads to slower decays of the relaxation of fluctuations which are often modeled by empirical rheological power-laws [12,13]. One systematic way of dealing with these types of memory effects is to replace the first order time derivative in the conventional hydrodynamic (transport) equations by a fractional time derivative which is interpreted as the memory or the after-effect of the underlying stochastic process [14]. These effects on correlation functions have been studied for some complex fluids [9,14,15].

The purpose of this work is to study and compare the effect of time fractional derivatives on the correlation function of the transverse velocity fluctuations of a (homogeneous) viscoelastic fluid by using a *GLE*. We consider a long-ranged (power-law) viscoelastic memory and a noise with a long-range (power-law) auto-correlation. More specifically, we first evaluate the transverse velocity fluctuations correlation function for conventional time derivatives and then introduce time fractional derivatives in the equation of motion of this correlation to calculate the corresponding fractional correlation function [16]. Since for finite frequencies the imaginary part of the dynamic shear modulus (loss modulus), $G^{\prime \prime }\left(\omega \right)$, of the dynamic viscosity η(ω) can be expressed in terms of the time-correlation function of the transverse velocity fluctuations, we compare the moduli for the non-fractional and fractional cases and find that the fractional modulus may be three times larger than the non-fractional one.

To this end the article is organized as follows. In Section 2 we set up the *GLE* for the dynamics of internal fluctuations of a viscoelastic fluid. Then an analytic exact expression for the one-time non-fractional correlation function for transverse velocity fluctuations (*NF*) is derived for power-law viscoelasticity. In Section 3 and Section 4, we introduce both, noise and fractional time derivatives into the *GLE* and the fractional temporal transverse velocity fluctuations correlation function, ${\hat{C}}_{F}\left(\vec{k},t\right)$, where $\vec{k}$ is the wavevector, is calculated analytically from them. We find that its magnitude is always lower than the non-fractional one and decays more rapidly. The relationship between $G^{\prime \prime }\left(\omega \right)$ and ${\hat{C}}_{F}\left(\vec{k},t\right)$ is calculated analytically. The difference between the values of $G^{\prime \prime }\left(\omega \right)$ for two specific viscoelastic fluids for accessible ranges of frequencies to our calculations is quantified. We find that the fractional effects on this measurable modulus may be as large as ~300% when compared with its non-fractional values. The fact that the dynamic shear modulus is related to the light scattering spectrum suggests that the measure of this property might be used as a suitable test to assess the effects of temporal fractional derivatives on a measurable property. Finally, in Section 5 we summarize the main results of our approach and emphasizes that the eventual validity of our model calculations can only come from experiments.

## 2. Model Formulation

The deformation of spatially homogeneous viscoelastic liquids near equilibrium is described by the linear response theory [17]. In this regime, the most general constitutive equation for the linear stress-strain relation is of the form [18],

(1) $$\sigma_{ij}\left(\vec{r},t\right)=-p\delta_{ij}+\int_{0}^{t}dt^{\prime }\left\{K\left(t-t^{\prime }\right){\dot{\gamma }}_{kk}\left(\vec{r},t^{\prime }\right)\delta_{ij}+2G\left(t-t^{\prime }\right)\left[{\dot{\gamma }}_{ij}\left(\vec{r},t^{\prime }\right)\right]-\frac{1}{3}{\dot{\gamma }}_{kk}\left(\vec{r},t^{\prime }\right)\delta_{ij}\right\}$$

The homogeneous character of these fluids comes from the assumption that the two independent, scalar moduli, the shear *G*(*t*) and the bulk (compressional) *K*(*t*), may only depend on time. Here, $\vec{r}$ is the position vector $\vec{r}=(x,y,z)$, $\sigma_{ij}\left(\vec{r},t\right)$ is the symmetric stress tensor, $p\left(\vec{r},t\right)$ is the pressure and $\left[{\dot{\gamma }}_{ij}\left(\vec{r},t\right)\right]$ is the rate of strain tensor, where the upper dot denotes the time derivative and Einstein’s summation convention for repeated indices is implied.

Consistency with linear response requires to linearize the hydrodynamic equations in the small deviations with respect to a reference equilibrium state identified by the subscript 0. This yields $\delta p\left(\vec{r},t\right)\equiv p\left(\vec{r},t\right)-p_{0}$ and $\delta v_{i}\left(\vec{r},t\right)\equiv v_{i}\left(\vec{r},t\right)$, where we have taken into account Galilei invariance for $\delta v_{i}$. The complete system of linearized hydrodynamic equations for δp and $\vec{v}$ then turns out to be [9,14,19],

(2) $$\frac{\partial }{\partial t}\delta \rho \left(\vec{r},t\right)=-\rho_{0}\frac{\partial v_{i}}{\partial x_{i}},$$

(3) $$\rho \frac{\partial }{\partial t}v_{i}\left(\vec{r},t\right)=-\frac{\partial \delta \rho }{\partial x_{i}}+\int_{0}^{t}dt^{\prime }\left\{\left[K\left(t-t^{\prime }\right)+\frac{1}{3}G\left(t-t^{\prime }\right)\right]\frac{\partial }{\partial x_{i}}\nabla \cdot \vec{v}\left(\vec{r},t^{\prime }\right)+G\left(t-t^{\prime }\right)\nabla^{2}\vec{v}\left(\vec{r},t^{\prime }\right)\right\}.$$

These equations are further simplified by choosing the direction of the z-axis as the longitudinal component and by separating $\vec{v}\left(\vec{r},t\right)$ into longitudinal ${\vec{v}}^{l}$ and transverse ${\vec{v}}^{t}$ components, $\vec{v}\left(\vec{r},t\right)={\vec{v}}^{l}\left(\vec{r},t\right)+{\vec{v}}^{t}\left(\vec{r},t\right)$, which are defined, respectively, by $\nabla \times {\vec{v}}^{l}=0$ and $\nabla \cdot {\vec{v}}^{t}=0.$ The linearized equations for the deviations are then

(4) $$\frac{\partial }{\partial t}\delta \rho \left(\vec{r},t\right)=-\rho_{0}\nabla \cdot v_{i}^{l}\left(\vec{r},t\right),$$

(5) $$\rho \frac{\partial }{\partial t}v_{i}^{t}\left(\vec{r},t\right)=\int_{0}^{t}dt^{\prime }G\left(t-t^{\prime }\right)\nabla^{2}v_{i}^{t}\left(\vec{r},t^{\prime }\right),$$

where *i* = *x*, *y*, identifies the two transverse components. In what follows we shall only consider one of them which will be denoted as $v\left(\vec{r},t\right),$ i.e., $v^{t}\left(\vec{r},t\right)\equiv v_{i=x}^{t}\left(\vec{r},t\right)\equiv v\left(\vec{r},t\right).$

### 2.1. Hydrodynamic Fluctuations

One of the simplest formulations to describe fluctuations in fluids near equilibrium is the *GLE* with additive Gaussian random forces [4]. According to this approach, the stochastic dynamic of v is described by

(6) $$\rho_{0}\frac{\partial }{\partial t}v\left(\vec{r},t\right)=\int_{0}^{t}dt^{\prime }G\left(t-t^{\prime }\right)\nabla^{2}v\left(\vec{r},t^{\prime }\right)+f(t),$$

where the additive fluctuating force f(t) is defined as a Gaussian, stationary, stochastic process with zero mean, 〈f(t)〉=0 and with a, so far, arbitrary (long range) correlation C(t)

(7) $$\langle f(t)f(t^{\prime })\rangle =C\left(\left|t-t^{\prime }\right|\right),$$

subject to the condition

(8) $$\langle f(t)v\left(\vec{r},t\right)\rangle =0.$$

Here, the angular brackets denote an average over both, the realizations of the noise and over an equilibrium ensemble of initial conditions. The rationale and an experimental evidence for assuming a Gaussian noise are as follows. It is well known that this noise describes the fluctuations around any equilibrium state and are dealt with in statistical mechanics with a variety of standard methods [5,20]. But when this state is (slightly) perturbed by changing the initial constraints in such a way that the state of the fluid remains within the linear response regime, the system relaxes to a new equilibrium state. The dynamics of the relaxation of the fluctuation is described by the *GLE*, Equation (6), and it is adequate to model them by a Gaussian process. This assumption has been experimentally shown to be appropriate for other complex systems involving the motion of tracers suspended in a fluid of swimming microorganisms [21]. In this system, the displacement of the tracers has a self-similar probability density function (pdf) with a Gaussian spatial effect which can be modeled by using a fractional diffusing equation [22,23]. On this basis, it seems reasonable to consider a Gaussian noise with a long-time correlation in Equation (6).

We define the combined Fourier–Laplace transform of an arbitrary field $A\left(\vec{r},t\right)$ by

(9) $$\tilde{A}\left(\vec{k},s\right)\equiv \int_{0}^{\infty }dte^{-st}\int_{-\infty }^{\infty }dte^{i\vec{k}\cdot \vec{r}}A\left(\vec{r},t\right),$$

where s=iω is the Laplace variable. In what follows, the caret $\left(\hat{A}\right)$ will denote its Laplace or Fourier transform with respect to one of its variables, whereas the tilde $\left(\tilde{A}\right)$ will indicate a transform with respect to both. Thus, from Equation (6) we get

(10) $$\langle \tilde{v}\left(\vec{k},s\right)\rangle =\frac{\rho_{0}}{\rho_{0}s+k^{2}\hat{G}(s)}\hat{v}(\vec{k}),$$

where $\hat{G}(s)$ is the Laplace transform of the so far arbitrary shear modulus G(t).

### 2.2. Power Law Viscoelasticity

There are many classes of materials in which the stress relaxation following a step strain is not close to an exponential, but is best represented by a power law in time, $G\left(t\right)\approx t^{-\beta }$. Examples of such materials—power law materials—include physically crossed-linked polymers, soft glassy materials and hydrogels. Non-exponential stress relaxation in the time domain also implies power law behavior in the viscoelastic storage modulus, $G^{\prime }(\omega )$, and in the loss modulus, $G^{\prime \prime }(\omega )$, measured in the frequency domain by using small-amplitude oscillatory shear deformations. This broad spectral response is indicative of the wide range of distinctive relaxation processes available to the microstructural elements that compose the material, and there is no single relaxation time [24]. Let us assume that the viscoelasticity of the fluid is well represented by a long-range power-law rheological equation of state, i.e.,

(11) $$G\left(t\right)=G_{0}t^{-\lambda },\;0<\lambda <1,$$

where $G_{0}$ denotes the zero-frequency shear viscosity. Then

(12) $$\hat{G}(s)=G_{0}\Gamma (1-\lambda )s^{\lambda -1},$$

where Γ(x) is the Gamma function. The parameter λ, measures the degree of viscoelasticity of the flow field; a low λ implies a weakly elastic flow field, whereas a large λ indicates an exceedingly elastic one.

### 2.3. Transverse Velocity Correlation

The single time auto-correlation function of the transverse velocity fluctuations is

(13) $$\hat{C}(\vec{k},t)\equiv \langle \hat{v}\left(\vec{k},t\right){\hat{v}}^{*}\left(\vec{k}\right)\rangle =\langle {\hat{v}}_{0}{\langle \hat{v}\left(\vec{k},t\right)\rangle }_{v_{0}}\rangle ,$$

where $v_{0}\equiv {\hat{v}}^{*}\left(\vec{k}\right)$ and the asterisk denotes complex conjugation. The notation indicates the following. Take a certain real constant value $v_{0}$ at t=0, calculate the average over the realizations of the noise f(t) conditional on the given $v_{0}$. Multiply it by $v_{0}$ and average this product over the values of $v_{0}$, as they occur in the initial equilibrium distribution. Therefore, from Equations (10) and (13) it follows that

(14) $$\tilde{C}(\vec{k},s)=\frac{\langle {\left|\hat{v}\left(\vec{k}\right)\right|}^{2}\rangle }{s+k^{2}\rho_{0}^{-1}\hat{G}(s)}.$$

Consistently with representing the viscoelasticity by Equation (11), we assume that the auto-correlation of the noise, Equation (7), is also a long-range power-law,

(15) $$\langle f(t)f(t^{\prime })\rangle =C_{\theta }t^{-\theta },\;0<\theta <1.$$

Then, for given $v_{0}$ Equation (10) yields

(16) $${\langle \tilde{v}\left(\vec{k},s\right)\rangle }_{v_{0}}=\hat{v}\left(\vec{k}\right)\frac{s^{\nu }}{s^{q}+b},$$

where $q\equiv 2-\lambda ,\;\nu \equiv 1-\lambda ,\;b\equiv \rho_{0}^{-1}G_{0}\Gamma (1-\lambda )k^{2}.$ The inverse Laplace transform of Equation (16) is well defined and is given by [25]

(17) $$L^{-1}\left(\frac{s^{\nu }}{s^{q}+b}\right)=R_{q,\nu }\left(-b,0,t\right)=R_{q,q-1}\left(-b,0,t\right)=E_{q}\left(-bt^{q}\right),$$

where $E_{q}\left(-bt^{q}\right)$ is the Mittag–Leffler function and $R_{q,\nu }\left(a,t\right)$ denotes the special function defined by the infinite series

(18) $$R_{q,\nu }\left(a,t\right)=\sum_{n=0}^{\infty }\frac{a^{n}t^{\left(n+1\right)q-1-\nu }}{\Gamma \left[\left(n+1\right)q-\nu \right]}.$$

Note that $R_{q,\nu }$ reduces to the exponential function $e^{at}$ when *q* = 1 and *ν* = 0 (see Equation (40) in Reference [25]), i.e., $R_{1,0}\left(a,t\right)=e^{at}$. Therefore, the conditional average is

(19) $${\langle \tilde{v}\left(\vec{k},s\right)\rangle }_{v_{0}}=\hat{v}\left(\vec{k}\right)E_{q}\left(-bt^{q}\right).$$

From Equations (13) and (19), it then follows that the normalized non-fractional (*NF*) transverse velocity correlation function for power-law viscoelasticity is given by

(20) $${\hat{C}}_{NF}(\vec{k},t)\equiv \frac{\langle \tilde{v}\left(\vec{k},t\right){\hat{v}}^{*}\left(\vec{k}\right)\rangle }{\langle {\left|\hat{v}\left(\vec{k}\right)\right|}^{2}\rangle }=E_{q}\left(-bt^{q}\right).$$

The explicit dependence of q≡2−λ and $b\equiv \rho_{0}^{-1}G_{0}\Gamma (1-\lambda )k^{2}$ on *λ* shows that the dynamics of ${\hat{C}}_{NF}$ is indeed affected by the viscoelasticity of the fluid.

**Figure 1:**
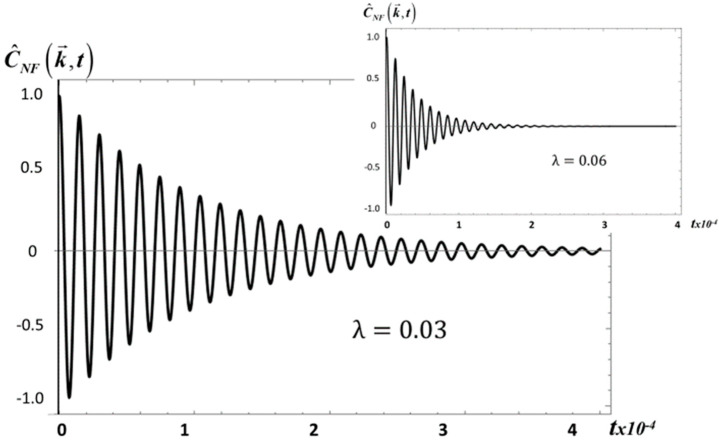
Plot of ${\hat{C}}_{NF}(\vec{k},t)$ as given by Equation (20), versus time (in seconds) *t*, for *S*_2_ with *λ* = 0.03 and 0.06. We choose the following material properties values: *T* = 295 K, *G*_0_ = 1.154 kg/ms, *ρ*_0_ = 971 kg/m^3^ [27].

To examine the behavior of Equation (20) quantitatively, we consider two specific viscoelastic fluids, namely, silicon oil (*S*_2_) and a solution of 0.02% separan MG500+2% water in glucose (*E*_1_). Chhabra et al. [26] have studied the rheological properties of these fluids and according to their shear stress and normal stress data, *S*_2_ would be classified as a weakly elastic fluid with a small *λ*, whereas *E*_1_ is exceedingly elastic and has a large *λ*. Figure 1 shows the plot of ${\hat{C}}_{NF}(\vec{k},t)$ as a function of time *t*, as given by Equation (20), for the small values *λ* = 0.03, 0.06, which would correspond to a fluid like *S*_2_.

**Figure 2:**
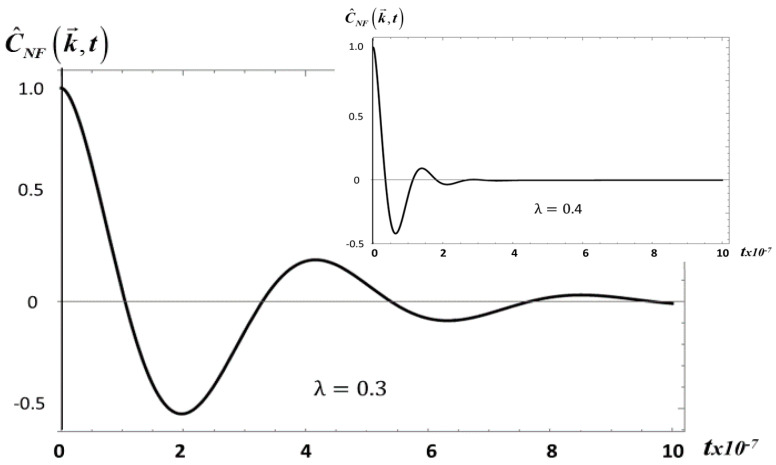
The non-fractional correlation ${\hat{C}}_{NF}(\vec{k},t)$ given by Equation (20), as a function of time *t* for *E*_1_ with *λ* = 0.3 and 0.4. Here *ρ*_0_ = 1414 kg/m^3^, *G*_0_ = 17.3 kg/ms, *T* = 292 K.

This figure shows that as *λ* increases and the viscoelasticity decreases, the amplitude and the range of the correlation also decrease. Actually, the same behavior is observed in Figure 2 for *E*_1_, as *λ* increases from *λ* = 0.3 to *λ* = 0.4. However, in this case ${\hat{C}}_{NF}(\vec{k},t)$ decays three orders of magnitude faster than for *S*_2_.

## 3. Time Fractional Derivatives

In Equation (6), we now replace *∂*/*∂*t by a Caputo left handed fractional time derivative (*LHCD*) $D_{0+}^{\mu }$ defined by [28,29,30],

(21) $$D_{0+}^{\mu }v\left(\vec{r},t\right)\equiv \frac{1}{\Gamma \left(m-\mu \right)}{\int_{0}^{t}\left(t-\xi \right)}^{m-\mu -1}v^{\left(m\right)}\left(\vec{r},\xi \right)d\xi ,$$

where *μ* is the order of the derivative, m is an integer such that m−1<μ<m and $v^{\left(m\right)}\equiv \partial^{m}v/\partial t^{m}$. The order *μ* of the fractional derivative should be chosen within the interval 0< *μ* <1 (and *m* = 1), because in this way, the integral in Equation (21) takes into account the contribution of the past values of this first order non-fractional derivative, and Equation (6) becomes

(22) $$\rho_{0}D_{0+}^{\mu }\left[v\left(\vec{r},t\right)\right]=\int_{0}^{t}dt^{\prime }G\left(t-t^{\prime }\right)\partial_{xx}^{2}v\left(\vec{r},t^{\prime }\right)+f\left(t\right).$$

After ensemble averaging and Fourier transforming with respect to *x,* this equation reads

(23) $$\rho_{0}D_{0+}^{\mu }\left[\hat{v}\left(k,t\right)\right]=-k^{2}\int_{t^{\prime }=0}^{t}G\left(t-t^{\prime }\right)\hat{v}\left(k,t^{\prime }\right)dt^{\prime }.$$

Since the Laplace transform of a Caputo derivative is given by [31]

(24) $$L\left\{D_{0+}^{\mu }f\left(t\right)\right\}=\frac{1}{s^{1-\mu }}\left[s\hat{f}\left(s\right)-f\left(0\right)\right]=s^{\mu }\hat{f}\left(s\right)-s^{\mu -1}f\left(0\right),$$

and since for the power-law viscoelasticity $\hat{G}\left(s\right)$ is given by Equation (12), from Equation (23) we obtain

(25) $$\langle \tilde{v}\left(k,s\right)\rangle ={\hat{v}}^{*}\left(k,0\right)\frac{s^{P}}{s^{Q}+b},$$

with

(26) Q=μ−λ+1, P=μ−λ=Q−1.

**Figure 3:**
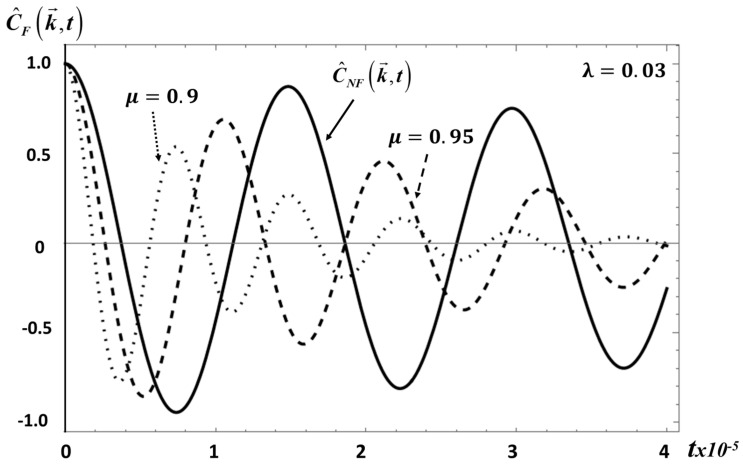
The transverse velocity correlation function ${\hat{C}}_{F}(\vec{k},t)$, as given by Equation (27), for *λ* = 0.03 and *μ* = 0.9 and *μ* = 0.95. The solid curve is ${\hat{C}}_{NF}(\vec{k},t)$ and is included for a reference comparison.

By using Equation (17) to invert Equation (25), we finally arrive at the following fractional time transverse velocity correlation function for power-law viscoelasticity

(27) $${\hat{C}}_{FT}(\vec{k},t)=E_{Q}\left(-bt^{Q}\right),$$

which is also given by a Mittag–Leffler function with different parameters. This correlation function is plotted in Figure 3 for *S*_2_ and for the same parameter values used in Figure 1.

**Figure 4:**
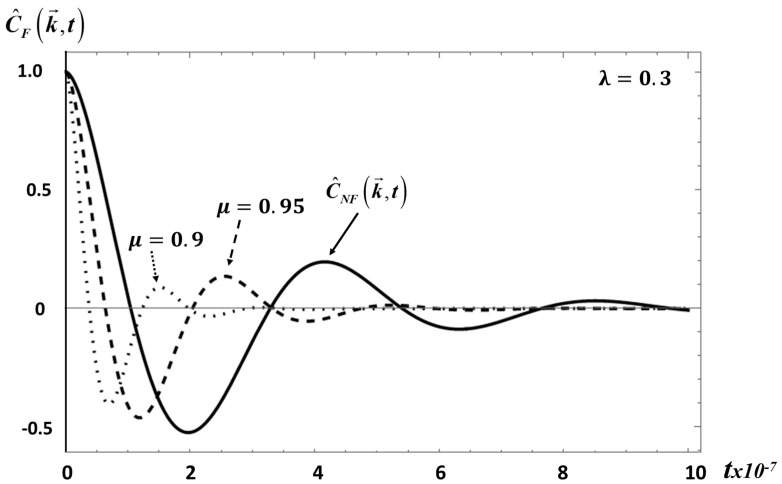
This figure shows the behavior of ${\hat{C}}_{F}(\vec{k},t)$, Equation (27), for *E*_1_ with *λ* = 0.3 and *μ* = 0.9, 0.95. Same material values as in Figure 2.

The behavior of ${\hat{C}}_{F}(\vec{k},t)$ for *E*_1_ is shown in Figure 4.

## 4. Dynamic Shear Modulus

If Equation (14) is compared with the corresponding one for a Newtonian fluid, namely [32],

(28) $${\tilde{C}}_{newt}(\vec{k},s)=\frac{\langle {\left|\hat{v}\left(\vec{k}\right)\right|}^{2}\rangle }{s+k^{2}\rho_{0}^{-1}\eta_{s}},$$

where *η_s_* is the shear viscosity, one can see that $\hat{G}\left(s\right)$ plays the role of a dynamic shear viscosity. It is useful to define the dynamic shear modulus as

(29) $$G*\left(\omega \right)\equiv i\omega \int_{0}^{\infty }dte^{-st}G\left(t\right)=G^{\prime }\left(\omega \right)+iG^{\prime \prime }\left(\omega \right),$$

where its real $G^{\prime }$ and imaginary parts $G^{\prime \prime }$ are given, respectively, by the sine-Fourier and cosine-Fourier transforms shown below. The real and imaginary parts of the dynamic viscosity η(ω) are related to $G^{\prime }\left(\omega \right)$ and $G^{\prime \prime }\left(\omega \right)$ by $\eta^{\prime }\left(\omega \right)=\omega^{-1}G^{\prime \prime }\left(\omega \right)$ and $\eta^{\prime \prime }\left(\omega \right)=\omega^{-1}G^{\prime }\left(\omega \right)$, respectively. Note that in the limit of vanishing frequency, *ω*→0, $G^{\prime }\left(\omega \right)\to 0$ and $\eta^{\prime \prime }\left(\omega \right)\to 0$, whereas $G^{\prime \prime }\left(\omega \right)\to 1$ and $\eta^{\prime }\left(\omega \right)\to \eta_{s}$. For finite frequencies $G^{\prime \prime }\left(\omega \right)$ or $\eta^{\prime }\left(\omega \right)$ can be expressed in terms of the time-correlation function of the transverse velocity by setting *s* = *iω* and by equating the imaginary parts of the equation to obtain [18]

(30) $$\frac{k^{2}}{\rho_{0}}G^{\prime \prime }\left(\omega \right)=\frac{\omega A\left(\vec{k},\omega \right)}{{\left[A\left(\vec{k},\omega \right)\right]}^{2}+{\left[B\left(\vec{k},\omega \right)\right]}^{2}},$$

where $A\left(\vec{k},\omega \right)$ and $B\left(\vec{k},\omega \right)$ are given, respectively, by

(31) $$A\left(\vec{k},\omega \right)=\int_{0}^{\infty }dt\cos\omega t\frac{\langle \hat{v}\left(\vec{k},t\right){\hat{v}}^{*}\left(\vec{k}\right)\rangle }{\langle {\left|\hat{v}\left(\vec{k}\right)\right|}^{2}\rangle },$$

(32) $$B\left(\vec{k},\omega \right)=\int_{0}^{\infty }dt\sin\omega t\frac{\langle \hat{v}\left(\vec{k},t\right){\hat{v}}^{*}\left(\vec{k}\right)\rangle }{\langle {\left|\hat{v}\left(\vec{k}\right)\right|}^{2}\rangle }.$$

It should be emphasized that Equation (30) provides a method of calculating $G^{\prime \prime }\left(\omega \right)$ if the time-correlation function of the transverse velocity can be calculated from a model, or if it can be measured by some experimental technique [18].

**Figure 5:**
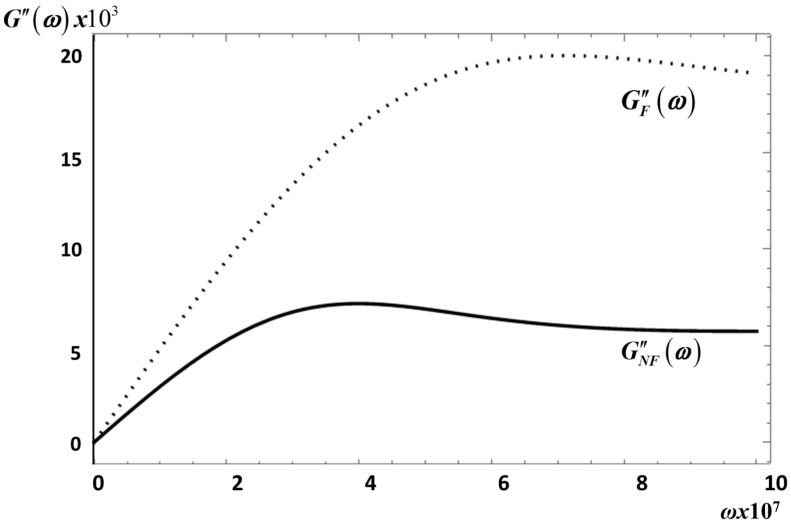
The non-fractional $G_{NF}^{\prime \prime }\left(\omega \right)$ (---) and fractional $G_{F}^{\prime \prime }\left(\omega \right)$ (...) loss moduli for *S*_2_ with *λ* = 0.05. Same material parameters as in Figure 1.

By inserting Equations (20) and (27) into Equations (31) and (32), and by substituting the result into Equation (30) we arrive at a complicated but analytic expressions for $G_{NF}^{\prime \prime }\left(\omega \right)$ and $G_{F}^{\prime \prime }\left(\omega \right)$, which we do not write down because it is not necessary for our purpose. For *S*_2_ this yields the solid curve for $G_{NF}^{\prime \prime }\left(\omega \right)$ and the dotted curve for $G_{F}^{\prime \prime }\left(\omega \right)$ in Figure 5.

**Figure 6:**
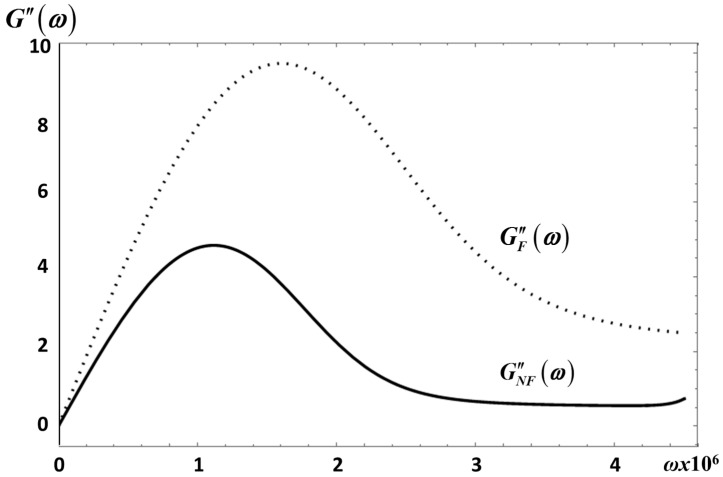
Behavior of $G_{NF}^{\prime \prime }\left(\omega \right)$ (---) and $G_{F}^{\prime \prime }\left(\omega \right)$ (...) for *E*_1_ with *λ* = 0.3. Same material parameters as in Figure 2.

For the frequency interval considered this figure shows that $G_{F}^{\prime \prime }\left(\omega \right)$ is always larger than $G_{NF}^{\prime \prime }\left(\omega \right)$. A feature of our model that shows that fractional effects in this modulus is a large effect that might be measurable. A similar behavior for these moduli is obtained for *E*_1_ as shown in Figure 6.

**Figure 7:**
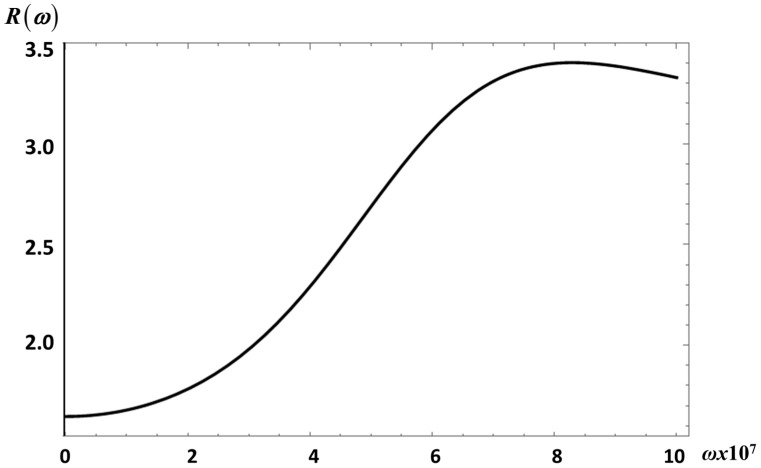
The ratio *R* vs. *ω* (s^−1^) for *E*_1_ as defined by Equation (33).

**Figure 8:**
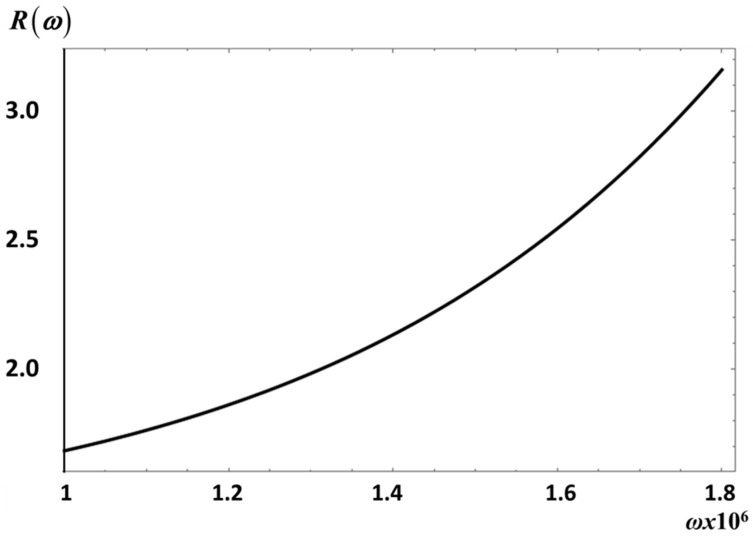
The ratio *R* vs. *ω* (s^−1^) for *S*_2_ as defined by Equation (33).

However, the difference between the fractional and non-fractional results can be better quantified if we plot the ratio *R*

(33) $$R\left(\omega ;\lambda ,\mu \right)=\frac{G_{F}^{\prime \prime }\left(\omega ;\lambda ,\mu \neq 1\right)}{G_{F}^{\prime \prime }\left(\omega ;\lambda ,\mu =1\right)}$$

of the fractional to the non-fractional moduli, as shown in Figure 7 and Figure 8, for *S*_2_ and *E*_1_, respectively.

The curve in Figure 7 shows R(ω;λ=0.3,μ=0.95) for *E*_1_; *R* is larger than one for the frequency intervals considered. Note, for instance, that *R* has a maximum value of about *R* ~ 3.4 at *ω* = 8.3 × 10^7^ s^−1^ and a minimum value of *R* ~ 1.63 for *ω* = 1 × 10^5^ s^−1^. This means that fractional fluctuations have a large effect on the transverse velocity fluctuations correlation than on the velocity correlation. These results indicate that the relative changes in *R*, are not a small effect and might be measurable. The same behavior of *R* is observed for *S*_2_ in Figure 8.

## 5. Discussion

In this work, we have analyzed the effects produced on the transverse velocity fluctuations correlation function ${\hat{C}}_{NF}(\vec{k},t)$, due to the presence of fractional temporal derivatives in the dynamics of these fluctuations. More specifically, we considered the case where the liquid possesses a long-range power viscoelasticity given by Equation (11) and the transport equations have long-range correlated stochastic terms. The interplay between the fluctuations arising from these two features motivated the introduction of fractional time derivatives into the hydrodynamic equations, since in this way it is possible to take into account the fact that the transverse velocity fluctuations evolve on vastly different time scales.

The most important results of our analysis are the analytic expressions for the different transverse velocity correlation functions, ${\hat{C}}_{NF}(\vec{k},t)$ and ${\hat{C}}_{F}(\vec{k},t)$ for the long-range power viscoelasticity and given, respectively, by Equations (20) and (27), both expressed in terms of Mittag–Leffler functions. Since for finite frequencies, $G^{\prime \prime }\left(\omega \right)$ can be expressed in terms of the time-correlation function of the transverse velocity, these fractional effects on the correlations do affect the frequency behavior of this measurable property of the system. To our knowledge, this issue has been scarcely considered in the literature of viscoelastic fluids.

Another issue that deserves further comments is the justification of the choice of the Caputo’s left-handed time derivative (*LHCD*), in spite of the fact that there exist other fractional time derivatives, such as those of Riemann–Liouville (*RL*) [29] or Grünwald–Letnikov [31]. The reason for choosing *LHCD* in this work lies in the physical initial conditions necessary to obtain a particular solution of Equation (22). When the *LHCD* is used in (22) to obtain its Laplace transform, the initial value $\hat{v}\left(k,0\right)$ and the initial value of the integer-order derivatives ${\hat{v}}^{\left(m\right)}\left(k,t=0\right)$, 1≤m≤α, should be known, but this is precisely the type of initial condition that can be specified or controlled in the relaxation process described by Equation (22); thus, it is consistent to use the *LHCD* in this equation. If we had used the *RL* time fractional derivative to obtain the Laplace transform (25), the initial values of the fractional derivatives $D_{0+}^{\mu }\left[v\left(\vec{r},t\right)\right]$ with β=α−k−1, k=0,1,…,n−1, where *n* is an integer such that n−1<α<n, would have to be known; however, for real physical systems like the viscoelastic liquid considered in this work, they are unknown. Thus, the *RL* fractional time derivative was discarded and the physically proper choice was the *LHCD* [33]. It should be remarked that for relaxation processes quite different from the study of the time evolution of fluctuations in viscoelasticity, other time fractional derivatives, different from that of Caputo, have been proposed. For example, in Ref. [34], a fractional anomalous-growth equation employing a Riemann–Liouville derivative has been introduced to describe nucleation-and-growth processes in superconducting materials like weakly viscoelastic cuprate systems where a relationship between the order of the fractional derivative and the fractal dimension of an anomalous random walk process was found. Another example is the use of Weyl derivatives to describe nucleation-and-growth processes in model lipid membranes [35]. In this latter example, a Caputo derivative of an order centering around ~1/2 is proposed to explain the plausible multitude of time scale of relevance. This form of introducing fractional derivatives has been also explored and its limitations have been discussed for a binary mixture in [33].

Finally, it should be emphasized that we are not aware of any specific experimental results to compare with the predictions of our model, and therefore, it is not possible to conclude from our analysis if this fractional behavior of various correlation functions and elastic moduli may be measurable; this is an open issue that remains to be assessed.
